# Ultrasensitive and Highly Selective Graphene-Based Single Yarn for Use in Wearable Gas Sensor

**DOI:** 10.1038/srep10904

**Published:** 2015-06-04

**Authors:** Yong Ju Yun, Won G. Hong, Nak-Jin Choi, Byung Hoon Kim, Yongseok Jun, Hyung-Kun Lee

**Affiliations:** 1 Department of Materials Chemistry and Engineering, Konkuk University, Republic of Korea; 2 Nano-bio Electron Microscopy Research Team, Korea basic Science Institute (KBSI), Daejeon, Republic of Korea; 3 R&D Team, Milaebo Co., Pyeongtaek, Republic of Korea; 4 Department of Physics, Incheon National University, Incheon, Republic of Korea; 5 Electronics & Telecommunications Research Institute (ETRI), Daejeon, Republic of Korea; 6 Advanced Devices Technology, Daejeon, Republic of Korea

## Abstract

Electric components based on fibers or textiles have been investigated owing to their potential applications in wearable devices. High performance on response to gas, drape-ability and washing durability are of important for gas sensors based on fiber substrates. In this report, we demonstrate the bendable and washable electronic textile (*e*-textile) gas sensors composed of reduced graphene oxides (RGOs) using commercially available yarn and molecular glue through an electrostatic self-assembly. The *e*-textile gas sensor possesses chemical durability to several detergent washing treatments and mechanical stability under 1,000 bending tests at an extreme bending radius of 1 mm as well as a high response to NO_2_ gas at room temperature with selectivity to other gases such as acetone, ethanol, ethylene, and CO_2_.

Fiber-shaped electric components such as thin film transistors (TFTs)^1^,^2^,^3^, light-emitting diodes (LEDs)^4^, energy harvesters^5^, energy storage devices^6^,^7^,^8^, and sensors^3^,^9^,^10^ with high performance and flexibility are of great interest owing to their potential applications in portable devices and wearable electronics. Fiber-based gas sensors are expected to have a large number of advantages including excellent drapability, pliability, durability, a light weight, and low cost^11^. Moreover, compared with conventional solid-state gas sensors or recently explored gas sensors prepared on flexible film or paper, fiber-based gas sensors can be integrated into textiles in various forms^12^. Thus far, several fiber sensors based on conducting polymers and metal oxides have been developed^13^,^14^,^15^. However, these sensors are unsuitable for flexible and wearable gas sensors owing to their poor mechanical/chemical durability and operation at high temperature. As an alternative approach, graphene and its derivatives have been the focus of research owing to their ultra-high surface area, excellent electrical properties, and good selectivity without the need for a catalyst or modification^16^,^17^. Additionally, they show high mechanical strength with flexibility^18^,^19^, which makes them ideal candidate materials for high-performance flexible gas sensors^20^,^21^,^22^,^23^.

## Results

**Figure 1:**
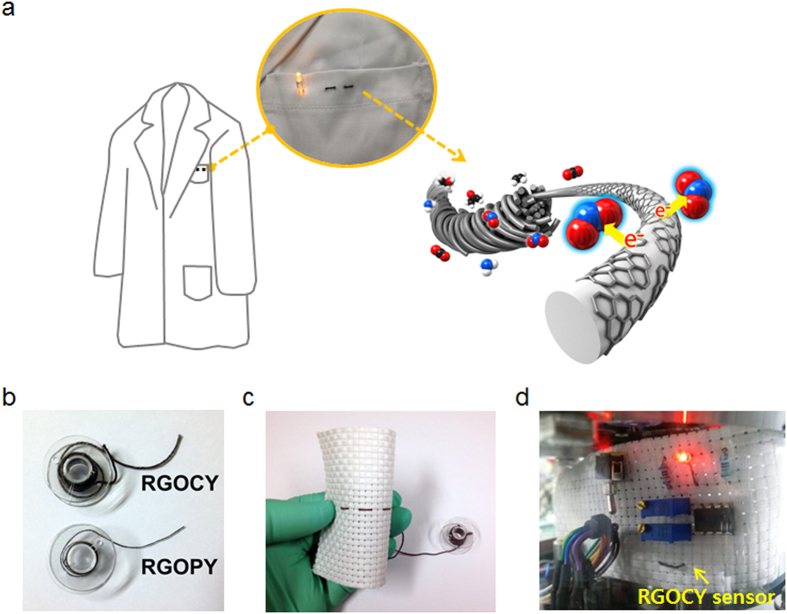
Fabric gas sensor with reduced graphene oxide applied. (**a**) Schematic illustration of RGOY gas sensor prepared from microfiber bundles (light grey cylinders) wrapped with RGO (dark grey hexagon patches). The red, blue, black, and white spheres indicate oxygen, nitrogen, carbon, and hydrogen atoms, respectively. (**b**) Photograph of RGOCY and RGOPY wound on a plastic bobbin. (**c**) RGOCY gas sensor system integrated into a fabric. (**d**) Demonstration of wearable gas sensing and alarm system. All parts of this figure were drawn and photographed by the authors.

Recently, graphene fibers and yarns have been developed for various applications, such as fiber-based supercapacitors, dye-sensitized photovoltaic cells (DSSCs), and photo-detectors^24^,^25^,^26^. However, there have been no reports to date on graphene yarn-based gas sensors. We previously reported an efficient graphene oxide (GO) wrapping method using biomolecules, i.e., bovine serum albumin (BSA), as a mediator for electrostatic self-assembly between the GO and various types of yarn, including cotton, polyester, and nylon^27^. This method was found to be effective for the preparation of ultrasensitive gas sensors and pliable electronic wires owing to their highly flexible and conducting properties. Herein, we demonstrate the effectiveness of wearable gas sensors based on reduced graphene oxide-decorated yarn (RGOY) with ultra-sensitivity at room temperature using a robust fiber wrapping method (Fig. 1a). RGOY gas sensors including RGO-decorated cotton yarn (RGOCY) and RGO-decorated polyester yarn (RGOPY) were obtained from GO wrapping through an electrostatic self-assembly using BSA and a low-temperature reduction. RGOCY and RGOPY 1-m long can be wound on a bobbin without damage (Fig. 1b), and were embroidered into commercial textile fabric and connected with electronic components to demonstrate a gas sensor device that turns on an LED light upon exposure to a certain gas concentration (Fig. 1c,d). These sensors can selectively detect NO_2_ gas down to 250 parts-per-billion (ppb) at room temperature with a –6.0% response, which is a three-fold higher response compared with an RGO gas sensor prepared on a flat substrate^28^. Furthermore, the sensors exhibit high reliability under several washing treatments and more than 1,000 bending tests with an extreme bending radius of as low as 1.0 mm, and provide a constant response for long-term (seven days) gas monitoring.

**Figure 2:**
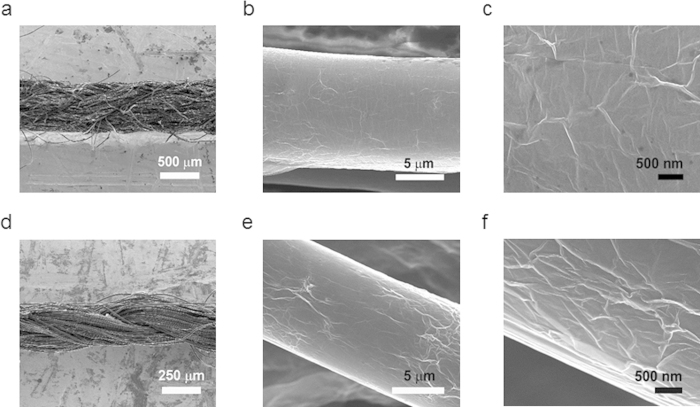
SEM images of RGOCY and RGOPY. (**a, d**) SEM images of single RGOCY and RGOPY (scale bar = 500 and 250 \micro m). (**b, e**) SEM images of RGOCY and RGOPY micro fibrils (scale bar =5 \micro m). (**c, f**) HR-SEM images of micro fibril surface of RGOCY and RGOPY (scale bar = 500 nm). Numerous wrinkles were observed, indicating that RGO is well-wrapped around the yarn.

Both cotton yarn (CY) and polyester yarn (PY) were used as templates for fabricating yarn wrapped with RGOs. Briefly, a dip-coating process was employed to coat an adhesive thin layer of BSA on the surface of both types of yarn. The resulting BSA-coated yarn (BSAY) was then wrapped with GO sheets through electrostatic self-assembly. Finally, graphene oxide yarn (GOY) was reduced using a low-temperature chemical reduction method. More details regarding the fabrication of RGOCY and RGOPY through a flow process can be found in our previous work, and are provided in the supporting information (Fig. S1)^27^. After chemical reduction, the color of the CY and PY changed from white to black. SEM images of RGOCY and RGOPY composed of single RGO microfibers with a diameter of 10 μm are shown in Fig. 2. The enlarged images (Fig. 2c,f) show numerous wrinkles and ripples around the individual fibers, which are attributed to the successful wrapping of the RGO sheets onto the CY and PY.

The RGOY was further characterized using Raman spectroscopy and X-ray photoelectron spectroscopy (XPS). The Raman spectra of pristine yarn, BSAY, GOY, and RGOY were compared, the results of which are shown in Fig. S2. After reduction of the GOY, the I_D_/I_G_ ratio in the Raman spectrum (black line) of the RGOY (RGOCY and RGOPY) was increased compared with that of the GOY (brown line). These results indicate that GOY is chemically converted into RGOY. This chemical evolution was also confirmed through a diminished O 1 s/C 1 s ratio and based on the reduced intensity of the oxygen-containing groups in the XPS C 1 s core-level spectra as compared to GOY (Fig. S3)^29^,^30^.

**Figure 3:**
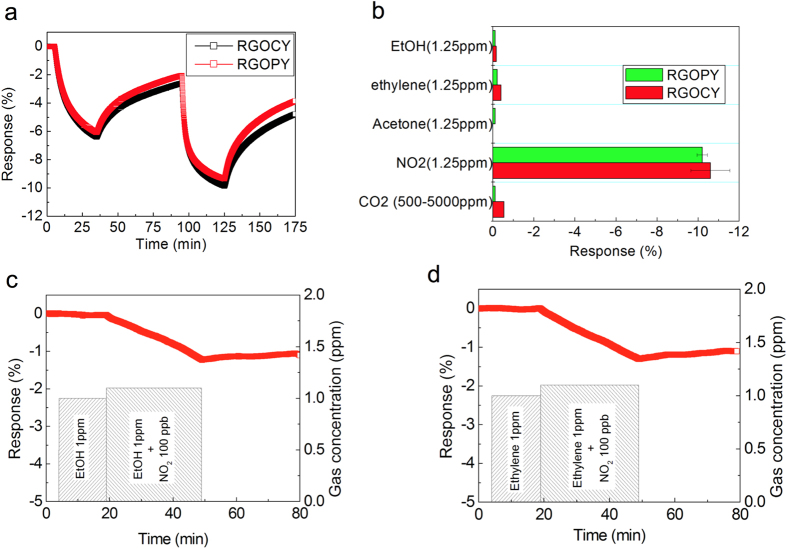
(**a**) Gas sensing performance of RGOCY and RGOPY exposed to NO_2_ gas of 0.25 ppm and 1.25 ppm at room temperature. (**b**) Gas response of RGOCY and RGOPY exposed to various gases at a concentration of 1.25 ppm. (**c**) Gas response of RGOCY to 100 ppb NO2 under the existence of 1 ppm EtOH. (**d**) Gas response of RGOCY to 100 ppb NO2 under the existence of 1 ppm ethylene.

The gas sensing behavior of RGOCY and RGOPY was investigated for various gases, including ethanol (EtOH), acetone, ethylene, CO_2_, and NO_2_, at room temperature as the working conditions. The sensor response (*R*) was defined as *R* (%) = (*R*_*g*_–*R*_*a*_)/*R*_*a*_ × 100, where *R*_*g*_ and *R*_*a*_ denote the electrical resistance upon exposure of analyte gas and air, respectively. When a sensor composed of reduced graphene oxide as a *p*-type semiconductor was exposed to NO_2_ as an oxidizing gas, the resistance, *R*_*g*_, of the sensor decreased owing to the increased hole concentrations resulting in the negative sign of the response^23^,^31^. The responses of RGOCY and RGOPY were –7.0% and –6.0% upon exposure to 0.25 ppm NO_2_ for 30 min (Fig. 3a). RGOCY and RGOPY showed a –12% response to NO_2_ gas at 1.25 ppm at room temperature, which is a very sensitive behavior to NO_2_ gas when compared with the –12% response of a micro-porous graphene form to 200 ppm NO_2_ gas^32^. However, the response and recovery time of the sensor is over a few hours. Carbonous materials such as carbon nanotube and graphene derivatives show strong affinities to NO_2_ gas. Appliance of an external energy such as UV irradiation or heat has been known as an effective method to facilitate desorption of the gas molecules on the surface of the sensing material, which helps the sensor composed of carbonous material to regenerate sensing properties^33^. Furthermore, RGOCY and RGOPY show high responses to NO_2_ gas at 1.25 ppm, but show very small responses when exposed to other gases including EtOH, acetone, ethylene, and CO_2_. The high response to NO_2_ and low responses to other gases have been known as an advantage of carbon-based materials as sensing materials (Fig. 3b)^34^. The sensor composed of RGOCY gave a response to 100 ppb NO_2_ gas even with co-existence of interferents such as 1 ppm EtOH or 1 ppm ethylene. RGOCY sensor did not respond to these interferents but respond to 100 ppb NO_2_ gas that was 10 times dilute concentration compared to those of interferents (Fig. 3c,d). These competitive sensing experiments confirm that the sensor device can discriminate dilute NO_2_ gas from concentrated ethanol or ethylene gas.

**Figure 4:**
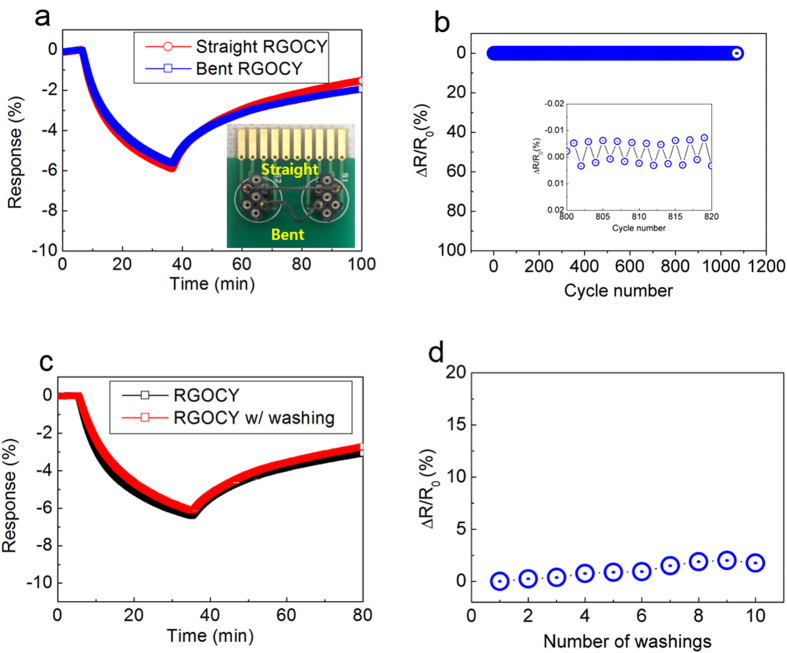
Twisting and washing tests for wearable gas sensors. (**a**) Gas response of straight and twisted RGOCY exposed to NO_2_ at 0.25 ppm. Inset photographs show the two samples (upper, straight; lower, bent). (**b**) Electrical resistance change of RGOCY upon repeated bending and straightening to a radius of 1 mm for over 1,000 cycles. (**c**) Gas response of RGOCY exposed to NO_2_ at 0.25 ppm after washing with a detergent. (**d**) Electrical resistance of RGOCY and RGOPY based on the number of washing treatments.

Wearable devices need to be robust because they will be exposed to mechanically demanding environments during cloth fabrication and the use of smart textiles in daily life. Therefore, their gas-sensing and electrical properties under various mechanical stresses such as bending and washing need to be clarified. Sensing behaviors depending on morphological shapes such as straight and bent forms were investigated during exposure to 0.25 ppm NO_2_ gas at room temperature (Fig. 4a). We found that straight RGOCY shows a similar response to that of twisted RGOCY owing to the bendable and flexible natures of CY and RGO, respectively. There have been a number of flexible gas sensors based on flat substrates, such as polyimide^23^, PDMS^35^, and paper^36^,^37^. Such flexible gas sensors have shown limited or comparable sensitivities compared to gas sensors based on rigid substrates. Interestingly, RGO-wrapped yarn was found to be highly sensitive to analyte gas under even twisted or bent formations. Bending tests were conducted using a custom-built two-point bending device and a high-precision mechanical system (Fig. S4a). Although the RGOCY became bent with a bend radius of 1.0 mm from position 1 to position 7, the electrical resistance remained approximately constant compared to that of RGOCY under a straight formation, which indicates the excellent durability of the samples under bending conditions. After 1,000 consecutive bending-straightening cycles, RGOCY and RGOPY showed excellent electrical conductivity retention (Figs. 4b, S4b, and S5a).

Durability to washing treatment is expected to be one of the important requirements for wearable devices. After washing RGOCY in a commercially available detergent solution using a magnetic stirrer, the sensor showed a marginally lower response than the non-treated RGOCY sensor (Fig. 4c). Stability under washing is related to the chemical resistance against water and synthetic detergent, as well as mechanical resistance against shearing resulting from friction between the yarn and magnetic stirrer. We found that there were no significant changes in the electrical conductivity after ten washing tests (Fig. 4d). The proposed RGOY sensor may be applied as a gas sensor in wearable devices owing to its high electrical resistance under chemical and mechanical stress, which is a necessary characteristic of a practical gas sensor.

**Figure 5:**
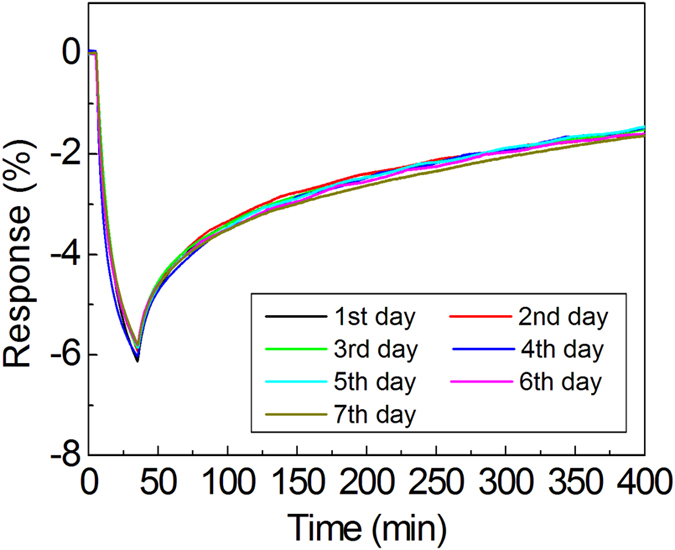
Gas sensing/recovery behaviors depending on the elapsed time during a one-week period. RGOCY exposed to NO_2_ at 0.25 ppm for 30 min shows a –6.0% response with a negative sign. The RGOCY responses remained constant with a 0.13% deviation during a one-week period.

The response of RGOCY to 0.25 ppm NO_2_ was monitored for a one-week period by exposing the RGOCY to NO_2_ for 30 min each day, to which the material showed a relatively constant response and recovery behaviors (Fig. 5). This long-term stability of RGOCY can be understood based on the robust wrapping of the yarn with RGOs, enabling the RGOCY to endure the stress resulting from the repeated adsorption and desorption of gas molecules on the surface of the RGOs. This superior performance shows the promise of RGOCY and RGOPY as materials for practical wearable electronics. An RGOCY sensor was weaved into a cotton fabric with an embedded electric circuit for turning on an LED light as an alarm upon exposure to 5.0 ppm of NO_2_ (Fig. S6 and Movie S1), the results of which demonstrate that RGOCY can be effectively weaved into commercial textile fabrics.

In conclusion, we demonstrated the fabrication of a bendable and washable gas sensor composed of reduced graphene oxide and commercially available yarn. The developed sensor possesses several remarkable features including chemical durability, mechanical stability, and a high response to NO_2_ gas. Most notable is its ultra-sensitivity as a gas sensor even at room temperature, which is made possible through robustly wrapping the yarn with RGOs, and by the large accessible surface area of yarn composed of several hundred fibrils. Such capabilities suggest the practical applications of the developed gas sensor for wearable and flexible electronics.

## Methods

### Preparation of RGOYs

Graphene oxide (GO) was prepared from natural graphite powder using a modified Hummers and Offenman method with H_2_SO_4_, NaNO_3_, and KMnO_4_^38^. For BSA functionalization, the commercially available as-obtained CY was soaked in a 0.5 wt% BSA solution for 30 min at room temperature. The resulting BSACY was then dried under a fume hood for 1 hr and washed with distilled water to remove any residual BSA molecules. The BSACY was then immersed in a bath containing 200 ml of a 2 mg/ml GO solution with mild shaking to enable the electrostatic self-assembly of the GO nanosheets onto the surfaces of the BSACY fibers. Upon completion of GO wrapping, the GOCY was dried under a fume hood for 1 hr. The RGOCY was reduced from GOCY using a hydriodic acid (HI) reduction method^29^. The as-prepared GOCY was immersed in a 100-ml glass beaker containing 2.0 ml of HI acid (57 wt% in H_2_O) and 5.0 ml of acetic acid ( >99.7%) at 40 °C for 10 min. Subsequently, the RGOCY was rinsed with a saturated sodium bicarbonate (NaHCO_3_) solution and water, and then dried at room temperature. Further details and a photograph of the RGOCY preparation are provided in the Supporting Information section (Figure S1). The RGOPY was prepared using the same procedure used to prepare the RGOCY.

### Measurement of the electrical and electromechanical properties of RGOY

The electrical conductivity was measured using a semiconducting parameter analyzer (Agilent 4154 A) under ambient conditions. The electromechanical stability of the samples as a function of the number of bending cycles was measured by repeatedly bending the yarn using a home-made two-point bending device. The 10-cm long fibers underwent straight-bending for over 1,000 cycles, and its electrical resistance was simultaneously recorded.

### Gas-sensing measurements

Sensor devices composed of 1.5 to 2.5-cm long RGOCY or RGOPY were placed in gas measurement chambers. Two chambers were used for measuring the sensor response. The first, a 300-cc volume chamber with a stainless steel wall and a single quartz observation window, was used for the response measurements of the single-yarn sensors. The other chamber, with a 2000-cc cylindrical shape and a quartz sidewall, was used for video recording the LED alarm upon exposure of the RGOCY sensor to NO_2_ gas. Air acting as a balance gas was used at a 1000-cc/min flow rate, whereas NO_2_ gas of 50 ppm was used as an analyte. The analyte was diluted with the balance gas to achieve the desired concentration of 0.25 ppm to 1.25 ppm using mass flow controllers (MFCs). The resistance changes of the sensors were recorded using an Agilent 34970 A digital multimeter^39^,^40^. Other analytes including acetone (50 ppm), ethylene (50 ppm), EtOH (50 ppm), and CO_2_ (10%) were diluted following the same experimental method used in the NO_2_ gas measurements.

## Additional Information

**How to cite this article**: Yun, Y. J. *et al.* Ultrasensitive and Highly Selective Graphene-Based Single Yarn for Use in Wearable Gas Sensor. *Sci. Rep.* **5**, 10904; doi: 10.1038/srep10904 (2015).

## Supplementary Material

Supplementary Information

Supplementary Movie 1
