# Supplementary material for: Ultrasensitive and Highly Selective Graphene-Based Single Yarn for Use in Wearable Gas Sensor
Source: Sci Rep. 2015 Jun 4;5:10904. doi: 10.1038/srep10904 (PMC4455253; doi:10.1038/srep10904)
Supplement: Supplementary Information [file srep10904-s1.pdf]

Supporting Information

Ultrasensitive and Highly Selective Graphene-Based Single Yarn for Use in Wearable Gas Sensor

*By Yong Ju Yun, Won G. Hong, Nak-Jin Choi, Byung Hoon Kim, Yongseok Jun, and Hyung-Kun Lee**

Experimental

Materials: Graphite powder (SP-1 graphite) was purchased from Bay Carbon (Michigan, USA). BSA powder, hydriodic acid (HI), acetic acid (CH_3CO_2H), formic acid (HCO_2H), sodium bicarbonate ($NaHCO_3$), sodium chloride (NaCl), sulphuric acid (H_2SO_4), sodium nitrate ($NaNO_3$), and potassium permanganate ($KMnO_4$) were purchased from Sigma-Aldrich (Germany).

Characterization: The thickness and lateral size of the dispersed GO nanosheets on a mica substrate were obtained using an atomic force microscope (AFM, Veeco, DI3100) with a sharp silicon probe (the radius of the tip curvature was < 5 nm). An SEM observation of the resulting RGOY was conducted using a field emission scanning electron microscope (FESEM, FEI Sirion 200). XPS measurements were taken using an ESCALAB 200R (Thermo VGScientific) with a monochromatic Al-Kα x-ray source at 250 W. Raman spectra were measured using a micro-Raman system (LabRAMHR, HORIBA scientific) with an excitation energy of 2.41 eV (514 nm).

Preparation process of RGOY

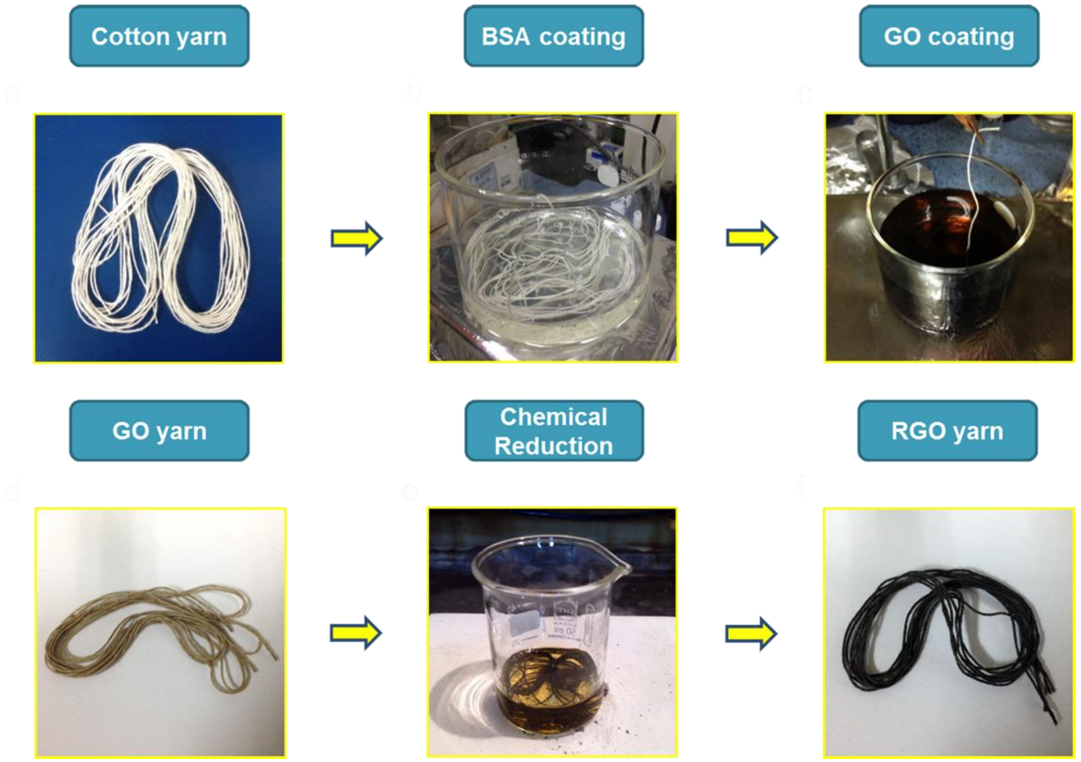

Figure S1. Photographs of the sesquential steps of the RGOY manufacturing process.

Raman spectroscopy of RGOY

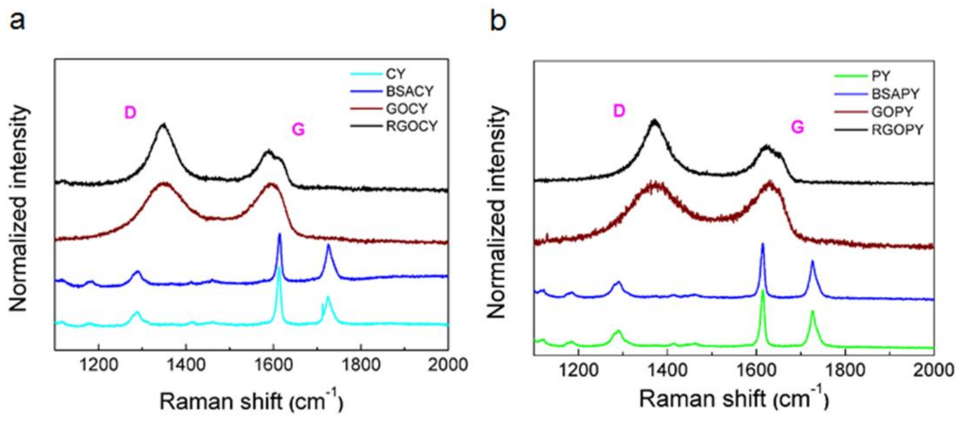

Figure S2. a, Raman spectra of CY (sky blue), BSACY (blue), GOCY (brown), and RGOCY (dark). b, Raman spectra of PY (green), BSAPY (blue), GOPY (brown), and RGOPY (black).

Figure S2 shows the change in Raman spectrum of the RGOY. The G peak at around 1,580 cm^{-1} arises from the stretching of the C-C bond of the sp^2 structure in both the rings and chains, whereas the D-peak at around 1,350 cm^{-1}, which is related to the breathing of the sp^2 carbon rings, is Raman active only in the presence of point defects [A. C. Ferrari, J. Robertson, Phys. Rev. Lett. 2000, 61, 14095].

XPS spectra of RGOY

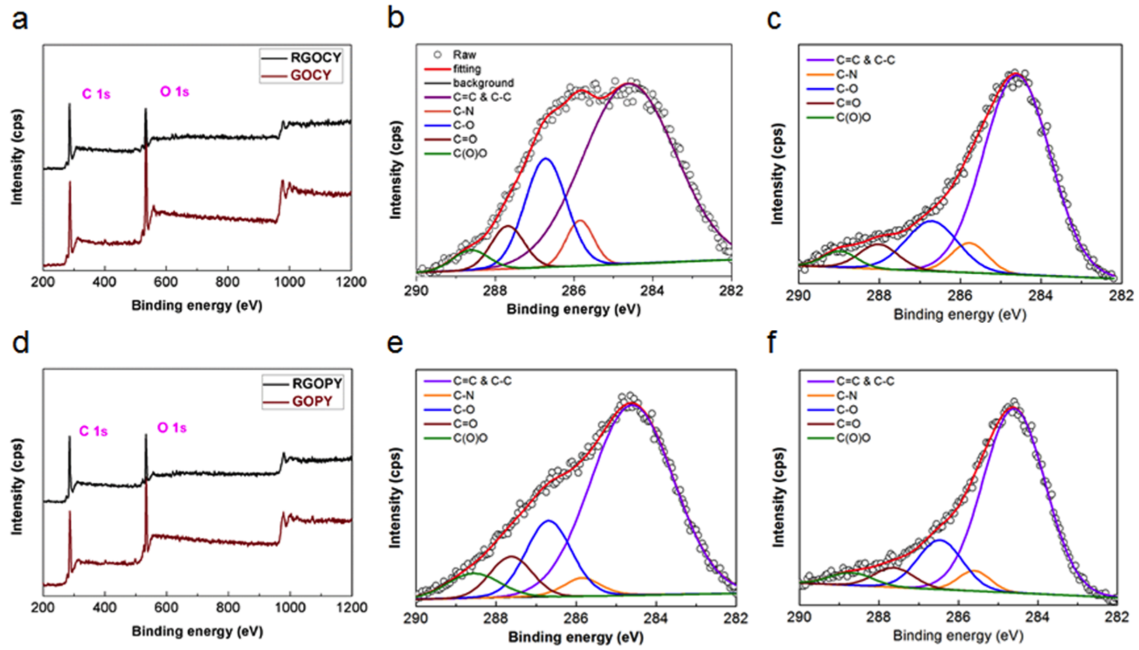

Figure S3. a, XPS survey of GOCY and RGOCY. b- c, High-resolution XPS C 1s core-level spectra of GOCY and RGOCY. d, XPS survery of GOPY and RGOPY. e-f, C 1s core-level spectra of GOPY and RGOPY.

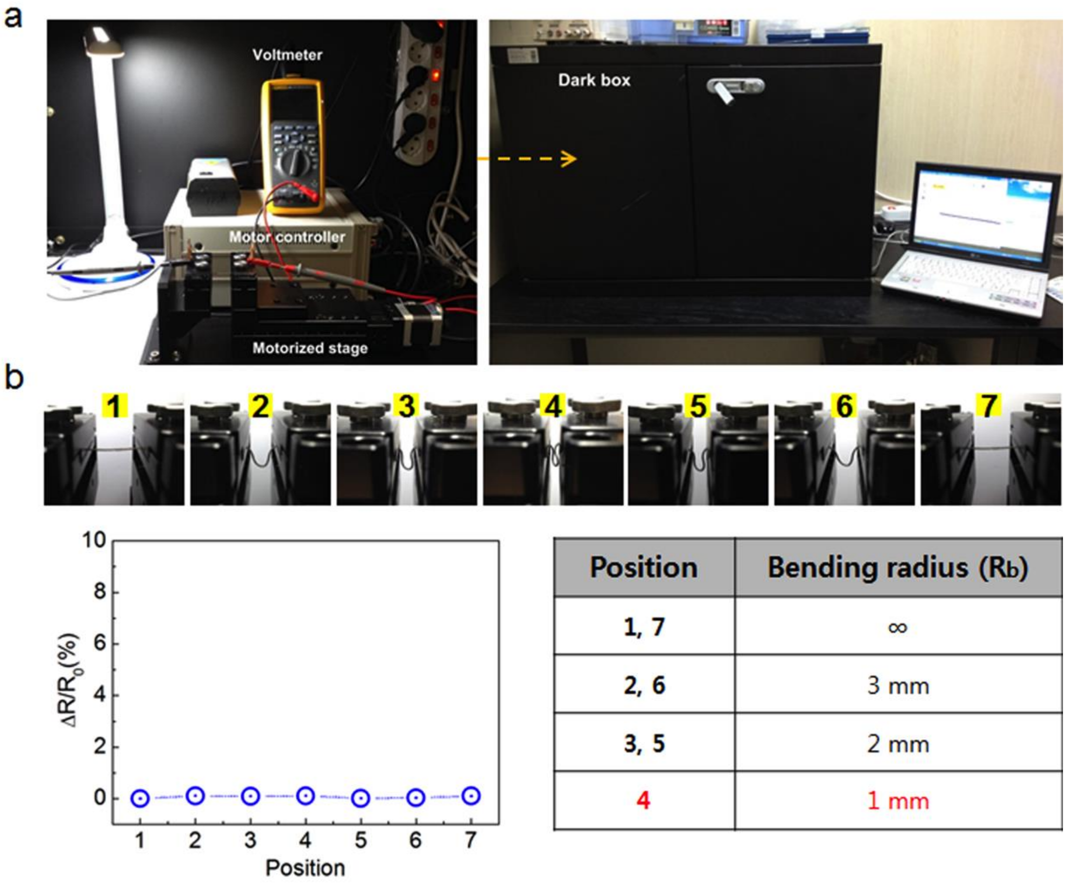

Figure S4. a, Cyclic bend testers for mechanical stability test of the gas sensors. b, Photographs and resistance change of RGOCY during the bending test.

Bending and washing tests of RGOPY

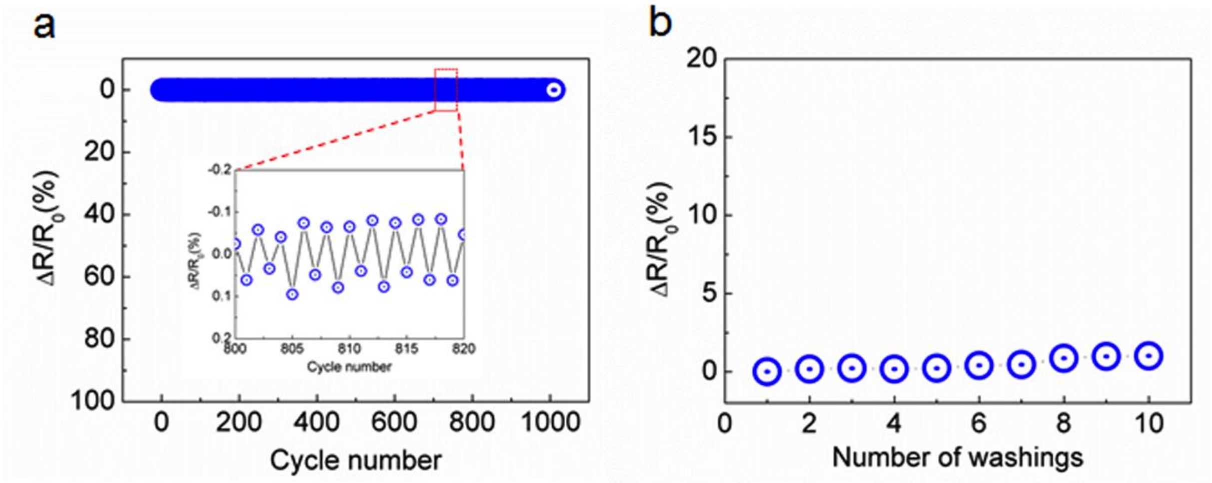

Figure S5. a, Electrical resistance change of RGOCY upon repeated bending and straightening to a radius of 1 mm for over 1,000 cycles. b, Electrical resistance of RGOPY as a function of the number of washing treatments.

RGOY gas sensor system

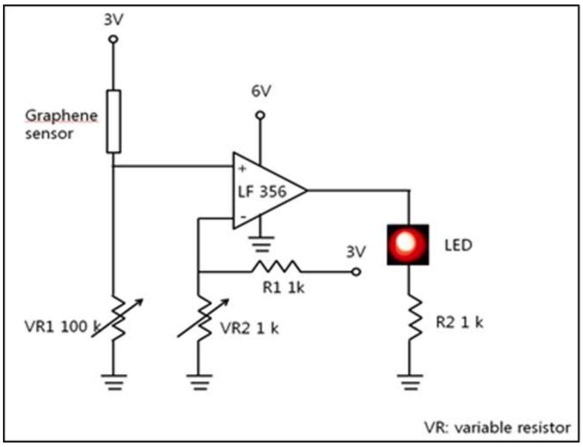

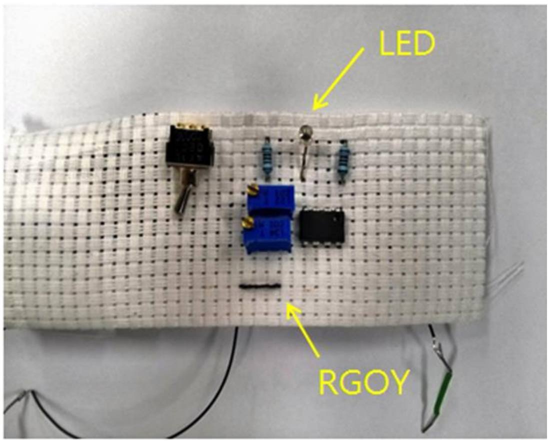

Figure S6. Equivalent circuit and optical image of RGOY gas sensor system.
